# SNX4 in Complex with Clathrin and Dynein: Implications for Endosome Movement

**DOI:** 10.1371/journal.pone.0005935

**Published:** 2009-06-16

**Authors:** Sigrid S. Skånland, Sébastien Wälchli, Andreas Brech, Kirsten Sandvig

**Affiliations:** 1 Centre for Cancer Biomedicine, Faculty Division Norwegian Radium Hospital, University of Oslo, Oslo, Norway; 2 Department of Biochemistry, Institute for Cancer Research, Norwegian Radium Hospital, Rikshospitalet University Hospital, Montebello, Oslo, Norway; 3 Department of Immunology, Institute for Cancer Research, Norwegian Radium Hospital, Rikshospitalet University Hospital, Montebello, Oslo, Norway; 4 Department of Molecular Biosciences, University of Oslo, Oslo, Norway; University of Birmingham, United Kingdom

## Abstract

**Background:**

Sorting nexins (SNXs) constitute a family of proteins classified by their phosphatidylinositol (PI) binding Phox homology (PX) domain. Some members regulate intracellular trafficking. We have here investigated mechanisms underlying SNX4 mediated endosome to Golgi transport.

**Methodology/Principal Findings:**

We show that SNX4 forms complexes with clathrin and dynein. The interactions were inhibited by wortmannin, a PI3-kinase inhibitor, suggesting that they form when SNX4 is associated with PI(3)P on endosomes. We further localized the clathrin interacting site on SNX4 to a clathrin box variant. A short peptide containing this motif was sufficient to pull down both clathrin and dynein. Knockdown studies demonstrated that clathrin is not required for the SNX4/dynein interaction. Moreover, clathrin knockdown led to increased Golgi transport of the toxin ricin, as well as redistribution of endosomes.

**Conclusions/Significance:**

We discuss the possibility of clathrin serving as a regulator of SNX4-dependent transport. Upon clathrin release, dynein may bind SNX4 and mediate retrograde movement.

## Introduction

Sorting nexins (SNXs) constitute a group of proteins classified by their phosphatidylinositol (PI) binding SNX-PX domain, a subgroup of the PX (Phox homology) domain family. Several of the SNXs have been shown to play a role in intracellular transport. For instance, SNX1 and SNX2 have been suggested to be part of the mammalian retromer [1]–[5], a coat complex involved in endosome to Golgi transport [6]. A few years ago, it was reported that there must be at least one additional retrieval pathway [7], and it was later shown that SNX4/41/42 mediate such a pathway in yeast [8]. SNX4 has also in mammalian cells been shown to mediate endosome to Golgi transport by a pathway different from SNX1 [9], [10].

SNX4 has a PI(3)P binding PX domain, and has been reported to localize to endosomes in an hVps34 dependent manner [9]. hVps34 is the PI3-kinase responsible for the production of most of the PI(3)P on endosomes [11]. In agreement with this, SNX4 is an effector of the hVps34 dependent endosome to Golgi transport pathway used by ricin [9]. This protein toxin is after endocytosis transported via early endosomes to the Golgi apparatus, and further to the endoplasmic reticulum (ER). From this compartment, it enters the cytosol where it exerts its toxic effect [12]. The role of hVps34 in ricin retrograde transport is in agreement with the suggestion that hVps34 activity may be required for minus-end-directed movement of endosomes along microtubuli [13]. This is further supported by the recent report showing that SNX4 is involved in endosomal sorting of the transferrin receptor via an indirect association with the minus end-directed microtubule motor protein dynein [10].

In the present study, we have identified clathrin heavy chain (CHC), tubulin and dynein as SNX4 interacting proteins. We have studied how these proteins interact, and characterized a novel clathrin box variant in SNX4. Upon CHC knockdown, Golgi transport of ricin is enhanced. We discuss the possibility of CHC serving as a regulator of SNX4-dependent transport. When CHC is released, dynein may bind SNX4 and mediate retrograde movement.

## Materials and Methods

### Reagents and antibodies

Ricin, wortmannin and nocodazole were from Sigma-Aldrich (St. Louis, MO). Restriction enzymes were from New England Biolabs (Ipswich, MA). Protein A Sepharose beads were purchased from Amersham Biosciences (Buckinghamshire, UK). Antibodies used in this study were anti-SNX4 (E-18; Santa Cruz Biotechnology, Santa Cruz, CA for WB and IP (2 µg per sample), and M01, clone 4H8; Abnova, Taipei City, Taiwan for IF), anti-clathrin heavy chain (CHC) (RDI Division of Fitzgerald Industries International, Concord, MA for WB, and Acris GmbH, Hiddenhausen, Germany for IF), anti-γ-tubulin and anti-α-tubulin (Sigma-Aldrich), anti-dynein (IC74; Chemicon International, Temecula, CA), anti-actin (Nordic Biosite, Täby, Sweden), anti-EEA1 (a gift from Harald Stenmark [14]), anti-myc (9E10; Santa Cruz Biotechnology), anti-ricin (Sigma-Aldrich), and anti-TGN46 (Serotec, Oxford, UK). HRP-conjugated secondary antibodies were from Jacksom Immunoresearch (West Grove, PA) and eBioscience (San Diego, CA).

### Cell culture

Human Embryonic Kidney (HEK) 293 cells were grown under 5% CO_2_ in DMEM supplemented with 10% fetal calf serum (FCS), 100 U/ml penicillin and 100 U/ml streptomycin (Invitrogen, Carlsbad, CA). The tissue culture plates were coated with Poly-L-lysine (Sigma-Aldrich) according to the manufacturer's protocol.

### Constructs, oligos and transfection

The wild-type SNX4 gene was a kind gift from Carol Haft (National Institutes of Health, Bethesda, MD) [15], and was amplified using the primers 5′-GCTCTAGACTATTACATCTTGCTAAAGCATTCCTT-3′ and 5′-TATAGATCTGAGCAGGCACCTCCGGAC-3′, which added *Xba*I and *Bgl*II restriction sites (underlined), respectively. The product was cloned into pEGFP-C1 (Novagen, Madison, WI) using these enzymes, resulting in GFP-SNX4. Full-length and mutant SNX4 with myc and 6×His tags were cloned using the primer 5′-TATAAGCTTATGGAGCAGGCACCTCCGGAC-3′ (both constructs), which added a *Hin*dIII restriction site, and 5′-ATACTCGAGCATCTTGCTAAAGCATTCCTT-3′ (SNX4 wt) or 5′-ATACTCGAGCTGAAACCCAGTTTCATTCAC-3′ (SNX4 198-451Δ), which added an *Xho*I restriction site. The product was cloned into pcDNA4 (Invitrogen) using these enzymes. SNX4 109-113Δ was generated from the wild-type using site-directed mutagenesis. All constructs were sequenced. GFP-CLC was a kind gift from Joshua Z. Rappoport (School of Biosciences University of Birmingham Edgbaston, Birmingham, UK) and James H. Keen (Thomas Jefferson University, Philadelphia, PA). Cells were transfected with the different constructs using Fugene 6 (Roche Diagnostics, Mannheim, Germany) according to the manufacturer's protocol.

To target clathrin heavy chain with siRNA, we used both vector and oligo based siRNAs. The validated targeting sequence in the vector was GCATGATGTGGTGTTCTTGAT (siCHCv) [16], and for the oligos GCAATGAGCTGTTTGAAGA (siCHCo) [17] and TAATCCAATTCGAAGACCAAT (siCHCo2) [18]. siRNAs against SNX4 were previously described [9]. Cells were transiently transfected for 3–4 days with plasmid DNA using Fugene 6 (Roche Diagnostics, Mannheim, Germany), or with 40 nM siRNA oligo for 3 days using Dharmafect 2 (Dharmacon, Lafayette, CO), according to the manufacturer's protocol. Control cells were transfected with a mock vector or a negative control oligo (Eurogentec, Seraing, Belgium), respectively.

### Immunoprecipitation

Cells were treated as indicated in the figure legends before lysis (lysis buffer: 0.1 M NaCl, 10 mM Na_2_HPO_4_ (pH 7.4), 1 mM EDTA, 1% Triton X-100, supplemented with a mixture of Complete protease inhibitors (Roche Diagnostics)). Cleared lysate was immunoprecipitated with the appropriate antibody, prebound to Protein A Sepharose beads, for the indicated time at 4°C. The immunoprecipitate was washed at least twice with 0.35% Triton X-100 in PBS and resuspended in sample buffer, subjected to SDS-PAGE and transferred to a PVDF membrane. Immunostaining was performed with the indicated antibodies. Alternatively, cells were lysed in the same lysis buffer containing 60 mM *n*-octyl-β-glucopyranoside, obtaining the same results (not shown).

### Confocal fluorescence microscopy

Cells were grown on glass coverslips and treated as indicated in the figure legends. The cells were fixed with 4% formalin solution (Sigma-Aldrich), permeabilized with 0.2% Triton X-100 in PBS and immunostained with appropriate antibodies. Fluorophore-labeled secondary antibodies were from Jackson Immunoresearch (West Grove, PA). DRAQ5 (Alexis Biochemicals, San Diego, CA) was used to stain the nuclei. The cells were mounted in Mowiol (Calbiochem, San Diego, CA) and examined by the laser scanning confocal microscope LSM 510 META (Carl Zeiss, Jena, Germany). Images were prepared and analyzed with the LSM Image Browser software (Carl Zeiss).

### Sulfation assays

Cells transfected as indicated were washed twice with sulfate-free medium (MEM 12–126, BIO Whittaker, Walkersville, MD) before incubation with 0.2 mCi/ml Na_2_^35^SO_4_ for 3 hours at 37°C in the same medium. They were next incubated with ricin sulf-1 [19] for 90 minutes. The cells were then washed with ice-cold PBS and lysed in lysis buffer (0.1 M NaCl, 10 mM Na_2_HPO_4_ (pH 7.4), 1 mM EDTA, 1% Triton X-100, supplemented with a mixture of Complete protease inhibitors). Ricin was immunoprecipitated from cleared lysate with anti-ricin antibodies. The immunoprecipitate was separated by SDS-PAGE and transferred to a membrane for investigation by autoradiography. Band intensities were quantified using ImageQuant 5.0 software (Molecular Dynamics, Sunnyvale, CA). As an internal control, total protein sulfation was measured after TCA-precipitation of the lysates, and shown to remain similar to control levels (data not shown).

### Mannosylation assays

Cells transfected as indicated were washed twice with glucose-free medium (DMEM 11966, Invitrogen) before incubation with 0.2 mCi/ml D-[2-^3^H(N)]-Mannose for 3 hours at 37°C in the same medium. Ricin sulf-2 [19] was then added, and the incubation continued for 3 hours. The cells were then washed with ice-cold PBS and lysed in lysis buffer (0.1 M NaCl, 10 mM Na_2_HPO_4_ (pH 7.4), 1 mM EDTA, 1% Triton X-100, supplemented with a mixture of Complete protease inhibitors). Ricin was immunoprecipitated from cleared lysate with anti-ricin antibodies. The immunoprecipitate was separated by SDS-PAGE and transferred to a membrane for investigation by autoradiography. Band intensities were quantified using ImageQuant 5.0 software (Molecular Dynamics, Sunnyvale, CA). As an internal control, total protein mannosylation was measured after TCA-precipitation of the lysates.

### GST protein purification and pull-down

Detailed description of construct cloning can be found in Text S1 online. GST-Hrs_707–775_ was a gift from Harald Stenmark [20]. Competent bacteria transformed with the different constructs were grown in 20 mL LB medium containing 50 mg/mL ampicillin at 37°C overnight. The next day, 180 mL LB/ampicillin was added, and the bacteria grown at 37°C until OD_600_ = 0.5. At this point, 1 mM IPTG (Saveen Werner AB, Malmö, Sweden) was added and the bacteria induced for 3 h at 37°C. The bacteria were pelleted by centrifugation at 4°C, and stored at −20°C until the next day. The pellet was then resuspended in 5 mL GST-buffer (20 mM Tris, pH 8.0, 100 mM NaCl) supplemented with complete protease inhibitors (Roche Diagnostics). The bacteria were next sonicated for 5 min on ice at 30% output. Triton X-100 (Sigma-Aldrich) was added to 1% final concentration and the lysate left for 30 min at 4°C with continuous rolling. A high-speed centrifugation at 4°C was performed, and the cleared lysate incubated with 100 µl Glutathione Sepharose beads (GE Healthcare, Uppsala, Sweden) in a sealed 10 mL disposable column (Bio-Rad, Hercules, CA) for 2 h at 4°C with continuous rolling. The lysate was then run through the column, and the beads washed once with 5 mL of each buffer 1 (0.1 M Tris, pH 7.8, 0.5 M NaCl), GST-buffer containing 0.35% Triton X-100 and GST-buffer alone. The column was sealed and the slurry containing GST-proteins resuspended in 100 µl GST-buffer supplemented with protease inhibitors and stored at 4°C.

In pull-down experiments, a similar amount of the different GST proteins were added to 20 µl Glutathione Sepharose beads (used as carrier) and 200 µl HEK293 cell lysate complemented with protease inhibitors. The mix was kept at 4°C overnight with rolling. The beads were washed 2–3 times with 0.1% Triton X-100 in PBS, and once with PBS alone. Reducing sample buffer was added, and the samples boiled at 95°C for 5 min before separation on SDS-PAGE and transfer to a PVDF membrane. Western blot was performed with the indicated antibodies.

## Results

### SNX4 interacts with clathrin, tubulin and dynein

**Figure 1:**
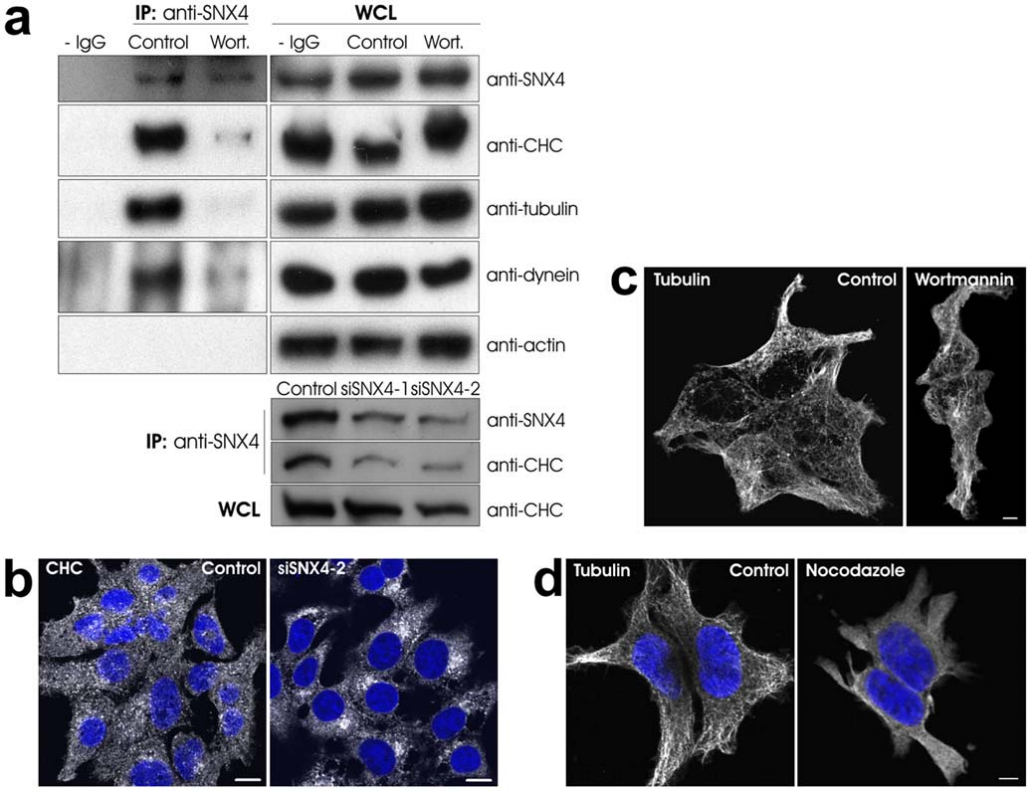
SNX4 co-immunoprecipitates CHC, tubulin and dynein. (a) HEK293 cells were treated with or without 100 nM wortmannin (Wort.) for 30 min, or transfected with control or SNX4 siRNA for 72 h, before lysis and anti-SNX4 immunoprecipitation at 4°C overnight. As a negative control, cell lysate was treated without antibody. The immunoprecipitate and whole cell lysate (WCL) were then separated by SDS-PAGE and analyzed by Western blot with the indicated antibodies. (b) Cells were transfected with control or SNX4 siRNA for 72 h before fixation and staining with anti-CHC antibodies. (c) Cells were treated with or without 100 nM wortmannin for 30 min before fixation and staining with anti-tubulin antibodies. (d) Cells were treated with or without 20 µM nocodazole for 30 min before fixation and antibody staining. DRAQ5 was used to stain the nuclei. Bars, 10 µm (b) and 5 µm (c, d).

In order to identify endosomal binding partners of SNX4, we performed co-immunoprecipitation experiments in the presence or absence of wortmannin, a PI3-kinase inhibitor shown to relocalize the PI(3)P binding SNX4 away from endosomes [9]. As shown in Figure 1a, clathrin heavy chain (CHC) was identified in the SNX4 precipitate, but not in the negative control (- IgG). A complex involving SNX4 and CHC has not previously been reported. Since we were interested in understanding how SNX4-positive endosomes move retrogradely, we also looked for the presence of cytoskeletal and motor proteins. We found that tubulin, but not actin, came down with SNX4 (Figure 1a). Furthermore, the minus end-directed microtubule motor protein dynein was identified (Figure 1a). This is in agreement with the recent finding that SNX4 interacts with KIBRA, a dynein-interacting protein [10], and suggests that SNX4 vesicle movement is mediated by dynein on microtubuli. The co-immunoprecipitation of SNX4 with CHC, tubulin and dynein was prevented in the presence of 100 nM wortmannin (Figure 1a), indicating that the interactions require membrane localization, most probably to the endosome [9]. As an additional negative control, cells were transfected with siRNA against SNX4, and the same experiment was performed. As expected, when SNX4 was knocked down, less CHC was co-immunoprecipitated with the remaining SNX4 (Figure 1a).

### Effect of wortmannin and nocodazole on cellular localization of CHC, tubulin and SNX4

Since SNX4 dissociates from endosomes upon wortmannin treatment [9], and the interaction between SNX4 and CHC, tubulin or dynein is lost at this condition (Figure 1a), we studied the cellular localization of the mentioned proteins upon PI3-kinase inhibition. In agreement with what was previously reported [20], we observed a more clustered distribution of CHC in the presence of wortmannin (not shown). Interestingly, a similar phenotype was observed upon SNX4 knockdown (Figure 1b). As expected, wortmannin did not affect the cellular distribution of tubulin (Figure 1c).

We next studied the effect of nocodazole, a microtubule depolymerising agent [21], on cellular protein localization. Tubulin was severely affected by nocodazole treatment (Figure 1d), as expected. At the same condition, less SNX4 staining could be observed (not shown). The latter could be due to reduced endosome size, as nocodazole has been reported to decrease the size of EEA1 positive vesicles [22]. However, nocodazole also affected the cell morphology (Figure 1d), which may lead to indirect effects on general protein localization.

### GFP-SNX4 co-localizes with tubulin and CHC

**Figure 2:**
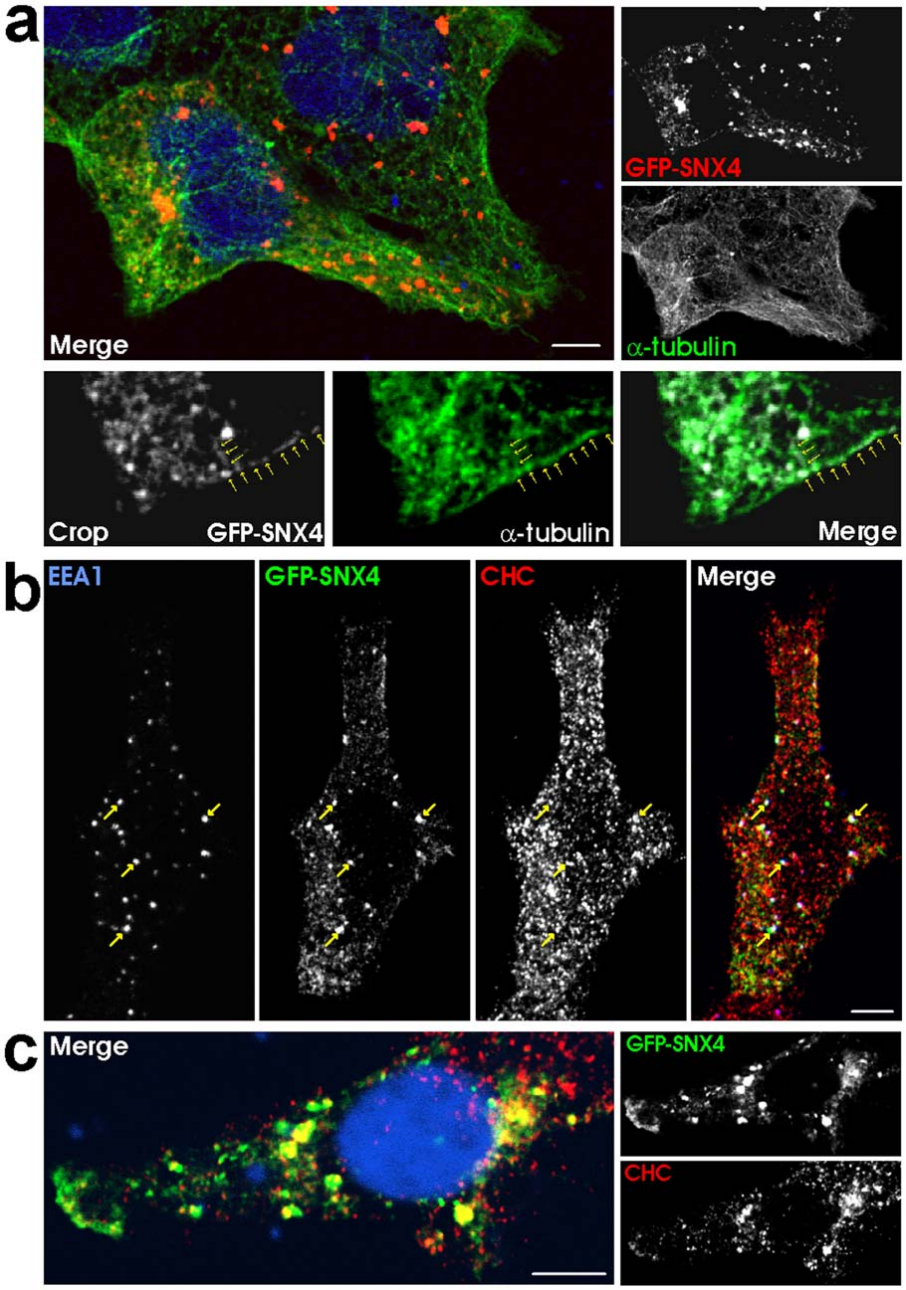
GFP-SNX4 co-localizes with tubulin and CHC. (a–b) Cells were transiently transfected with GFP-SNX4 for 24 h before the cells were fixed and stained as indicated. (c) Cells expressing high levels of GFP-SNX4 were fixed and stained as indicated. DRAQ5 was used to stain the nuclei. Bars, 5 µm.

Since the functional antibodies for use in immunofluorescence experiments were all raised in mice, we could not perform co-localization studies with endogenous SNX4 and its binding partners. To circumvent this, we cloned and expressed SNX4 as a GFP fusion protein. In agreement with the co-immunoprecipitation experiments, we observed GFP-SNX4 positive vesicles in close proximity to, or overlapping with, tubulin (Figure 2a). In the case of CHC, we observed a clear co-localization with GFP-SNX4, and even triple co-localization with the early endosomal protein EEA1 (Figure 2b). The fraction of GFP-SNX4 that co-localized with CHC at moderate expression level was quantified to be 27.8±6.6% (average with standard deviation, n = 15), and with tubulin 19.4±7.6% (average with standard deviation, n = 5). In order to exclude the possibility that this co-localization was random, we expressed GFP alone and performed the corresponding experiments. Even though GFP is expressed throughout the cell, less than 0.5% co-localization could be detected with CHC (n = 16) or tubulin (n = 10) (pictures not presented). This clearly demonstrates that the co-localization between GFP-SNX4 and CHC or tubulin is significant (p<0.0001 by Student's t-test). At high GFP-SNX4 expression level, CHC appeared more recruited to GFP-SNX4 positive vesicles (Figure 2c). We furthermore confirmed that endogenous SNX4 partly co-localized with transiently expressed GFP-CLC (clathrin light chain) (13.2±1.9%; average with standard deviation, n = 10) (Figure S1). These data support the suggestion of SNX4/tubulin and SNX4/CHC complexes on endosomes.

### SNX4 binds CHC via a clathrin box variant

**Figure 3:**
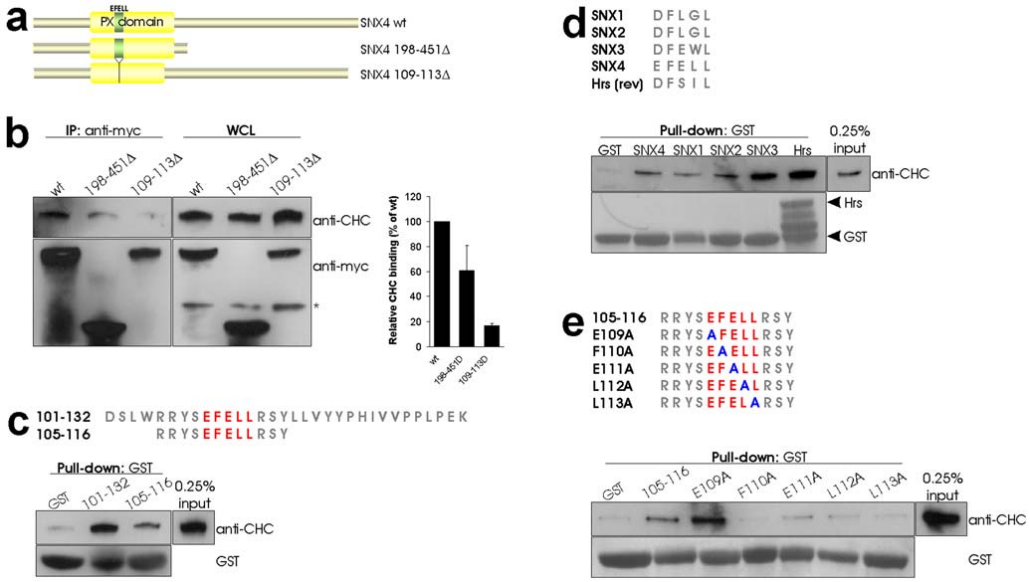
SNX4 interacts with CHC via a clathrin box variant. (a) Schematic view of SNX4 wt, SNX4 198-451Δ and SNX4 109-113Δ. (b) Cells transfected as indicated were lysed and proceeded to anti-myc immunoprecipitation for 4 h at 4°C. The immunoprecipitate and whole cell lysate (WCL) were separated by SDS-PAGE and analyzed by Western blot with the indicated antibodies. Quantification of CHC binding corrected for expression level of the myc constructs is shown to the right, n = 3. (c) The amino acid sequences of GST-peptides are shown with putative clathrin box in red. The numbering represents amino acid number in the SNX4 sequence. GST pull-down was performed with cell lysate at 4°C overnight. The pull-down was subjected to SDS-PAGE and analyzed by Western blot with the indicated antibodies. GST alone was used as negative control. (d) Putative clathrin boxes of SNX1, SNX2, SNX3, SNX4 (105–116) and reverse clathrin box of Hrs are aligned. GST pull-down was performed as in (c) with the indicated peptides. (e) Schematic view of GST-peptides containing the putative clathrin box (red) and point mutations (blue). GST pull-down was performed as in (c) with the indicated peptides.

A recognized CHC interacting motif is the so-called clathrin box LΦpΦ(−), where L is leucine, Φ a bulky hydrophobic residue, p a polar residue, and (−) is a negatively charged residue [23]. Amino acid sequence analysis of SNX4 did not show such a motif. However, a variant, or an “inverted” clathrin box, ^109^EFELL, was found in its PX domain. We therefore chose to test whether this sequence was required for CHC binding. We constructed the deletion mutant SNX4 109-113Δ and also designed a SNX4 construct deleted of its C-terminal half, SNX4 198-451Δ (Figure 3a). Both wild-type SNX4 and the two mutants were designed with a myc-tag. Since a mutation in the PX domain could potentially affect the endosomal localization of SNX4, we first investigated the cellular distribution of the recombinant SNX4 proteins by immunofluorescence. No apparent change in localization was observed for SNX4 109-113Δ compared to SNX4 wt (Figure S2a), but its co-localization with EEA1 was reduced to 67% relative to the wild type (n = 6 for SNX4 wt and 8 for SNX4 109-113Δ) (Figure S2b). SNX4 198-451Δ was for an unexplained reason present in the nucleus in addition to its expected cytoplasmic localization (Figure S2a). We next performed anti-myc immunoprecipitation experiments on cells expressing the SNX4 constructs, and examined the presence of CHC. As shown in Figure 3b, a mild reduction in the CHC binding was observed for SNX4 198-451Δ, whereas it was practically abolished for the SNX4 109-113Δ mutant. Three independent experiments were quantified and corrected for SNX4 expression level. The result showed an ∼80% reduction in CHC binding for SNX4 109-113Δ compared to SNX4 wt (Figure 3b). This suggests that the clathrin box variant present in SNX4 may be a CHC binding site. In order to validate this, we designed GST-peptides from the SNX4 sequence containing the putative CHC binding motif. We made one peptide containing amino acids 101–132, and a shorter one from amino acid 105–116 (Figure 3c). The peptides were purified and added to cell lysate before pull-down. CHC was present in both the 101–132 and 105–116 pull-downs (Figure 3c). GST alone was used as negative control and was unable to pull down CHC (Figure 3c). Sequences corresponding to ^109^EFELL in SNX4 are also present in other SNXs. We therefore investigated whether these sequences from SNX1, SNX2 and SNX3 could also pull down CHC. A sequence containing the clathrin box of Hrs was used as positive control [20]. The clathrin box of Hrs is represented from the C- to the N-terminal end (rev) and aligned with the putative clathrin boxes of the SNXs in Figure 3d. The main residues are conserved. In pull-down experiments all the peptides, but not GST alone, were able to bind CHC (Figure 3d). In order to study the contribution of the different amino acids of ^109^EFELL, we performed an “alanine scan” of the short peptide. We exchanged the five amino acids one by one with alanine (Figure 3e), and performed pull-down experiments with these peptides. We observed that the E109A mutant was still able to pull down CHC, perhaps more efficiently than the wild type, while the mutants F110A, E111A, L112A and L113A only showed background binding to CHC (Figure 3e). Interestingly, F110 and L113 are strictly conserved in all the putative clathrin boxes tested (Figure 3d), and might represent necessary residues. From these data we conclude that the SNX4 clathrin box variant is important for CHC binding.

### The SNX4 interaction with dynein does not require CHC

**Figure 4:**
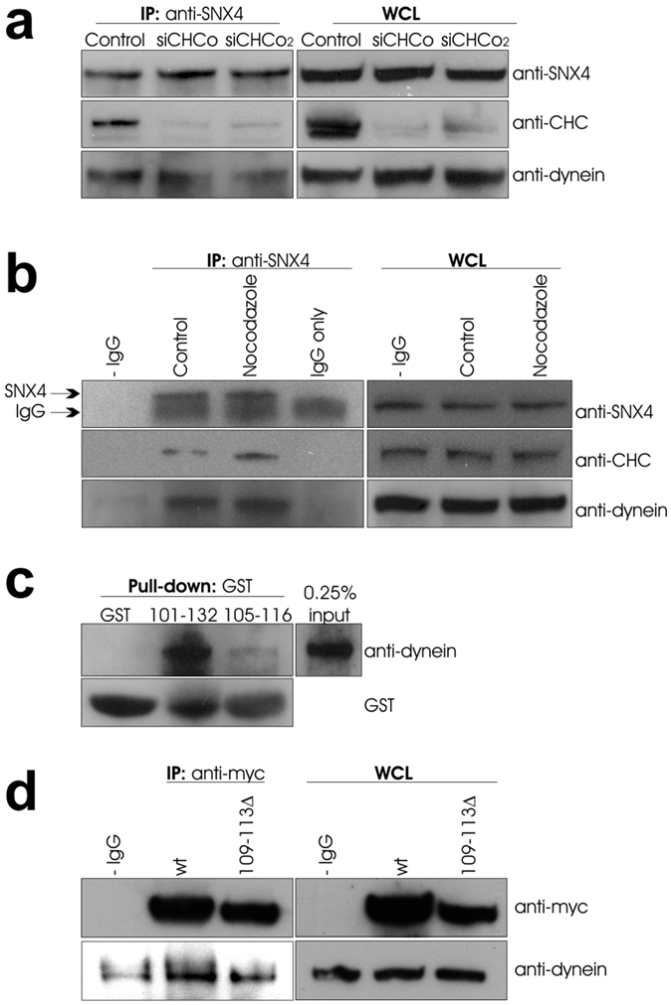
The SNX4 interaction with dynein does not require CHC. (a) Cells were transfected with control siRNA or siRNA targeting CHC (siCHC) as described in *Materials and Methods*. Lysate of the cells was then subjected to SNX4 immunoprecipitation at 4°C overnight, and the precipitate separated by SDS-PAGE before Western blot analysis with the indicated antibodies. (b) Cells were treated with or without nocodazole for 30 min before lysis and SNX4 immunoprecipitation. The precipitate was separated by SDS-PAGE and analysed by Western blot with the indicated antibodies. (c) GST pull-down of 101–132, 105–116 or GST alone was performed with cell lysate at 4°C overnight. The pull-down was subjected to SDS-PAGE and analyzed by Western blot with the indicated antibodies. (d) Cells transfected as indicated were lysed and proceeded to anti-myc immunoprecipitation for 3 h at 4°C. The immunoprecipitate and whole cell lysate (WCL) were separated by SDS-PAGE and analyzed by Western blot with the indicated antibodies.

To further investigate how SNX4 interacts with CHC and dynein, we used CHC siRNA to reduce the level of CHC, and then immunoprecipitated SNX4. If the SNX4/dynein interaction is independent of CHC, dynein should be found in the precipitate even when CHC is knocked down. As shown in Figure 4a, this was the case. This opens for the possibility that SNX4 forms separate complexes with CHC and dynein, an idea supported by the result that dynein and CHC bind to the same area of SNX4 (see below). We next investigated the effect of the microtubule depolymerising agent nocodazole on complex formation. As shown in Figure 4b, both CHC and dynein could still interact with SNX4 in cells treated with nocodazole. Interestingly, an increased binding of CHC to SNX4 was observed (2.30 fold±0.99, average±standard deviation, n = 3) (Figure 4b).

In order to clarify whether dynein and CHC bind to the same region of SNX4, we performed GST pull-down experiments with the peptides GST-SNX4 101–132 and 105–116 and probed with anti-dynein. Dynein bound to both peptides (Figure 4c), but the interaction was significantly stronger with the 101–132 peptide. This suggests that additional amino acids present only in the long peptide are involved in dynein binding, or that the additional amino acids give a better folding of this peptide. In order to test this, we performed immunoprecipitation experiments with the SNX4 109-113Δ mutant. As shown in Figure 4d, dynein could still interact with this mutant, indicating that the clathrin box alone is not sufficient for dynein binding.

### Knockdown of CHC causes increased retrograde transport of ricin

**Figure 5:**
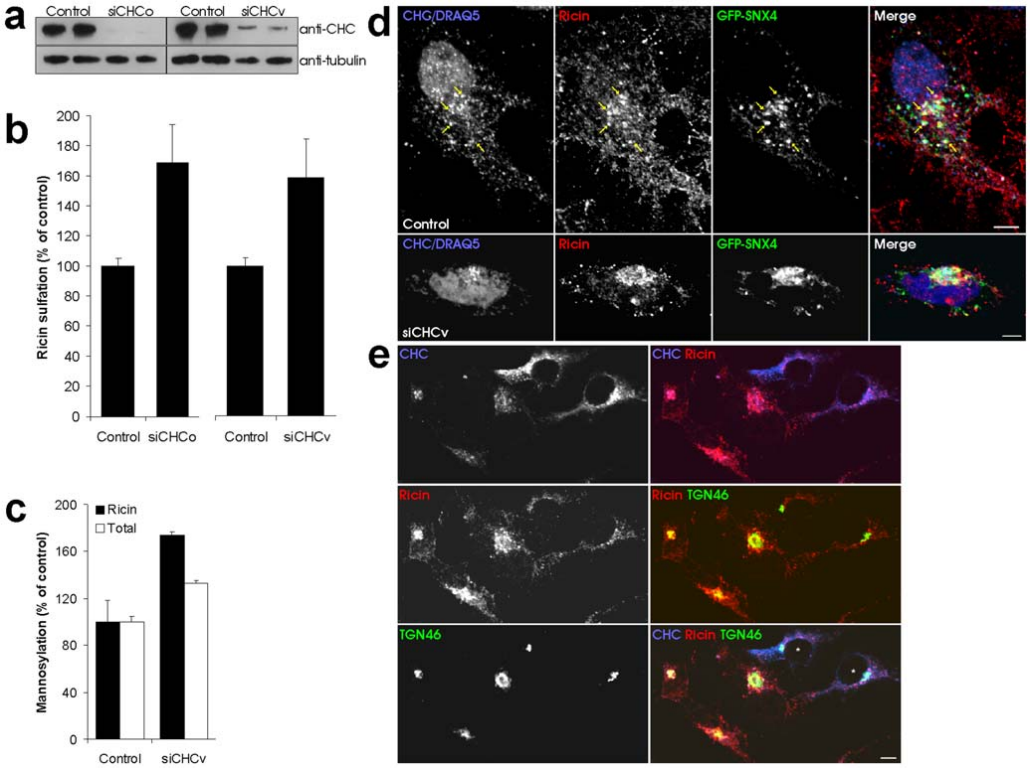
CHC knockdown leads to increased ricin transport. (a) Transfected cells were lysed and analyzed by Western blot with the indicated antibodies. (b) Cells transfected as indicated were incubated with radioactive sulphate for 3 h before further incubation with ricin sulf-1 for 90 min and subsequent lysis. Ricin was immunoprecipitated from the lysates, and the precipitate separated by SDS-PAGE before autoradiography. The intensity of the bands was quantified and the average plotted with error bars showing standard deviations. (c) Transfected cells were incubated with [^3^H]mannose in glucose free medium for 3 h before further incubation with ricin sulf-2 for 3 h. Cell lysate was immunoprecipitated with anti-ricin antibodies. The precipitate was analyzed by autoradiography. (d) Cells transfected as indicated were incubated with 2 µg/ml ricin for 45 min before fixation and staining with antibodies as indicated. Bars, 5 µm. (e) Cells transfected with siCHCv were incubated with 2 µg/ml ricin for 45 min before fixation and staining with the indicated antibodies. Asterisks in the merged picture indicate control cells. Bar, 5 µm.

Knockdown of SNX4 results in reduced endosome to Golgi transport of the protein toxin ricin [9]. Ricin is after endocytosis transported via early endosomes and the Golgi apparatus, to the endoplasmic reticulum (ER). Since we here show that SNX4 and CHC form a complex, we were interested in the effect of reduced CHC levels on ricin transport. We took advantage of siRNA to knock down CHC, and employed both the oligo-based and vector-based approach (siCHCo and siCHCv, respectively). Figure 5a shows the strong effect of these siRNAs on the protein level of CHC. One can measure the amount of ricin reaching the Golgi by studying the sulfation, a protein modification taking place only in the *trans*-Golgi network, of a genetically engineered ricin molecule containing a sulfation site [19]. Upon CHC knockdown, we observed increased ricin sulfation (Figure 5b). This is in contrast to what is seen with reduced SNX4 levels [9]. Importantly, several independent reports have shown that ricin endocytosis remains efficient in cells where clathrin-dependent endocytosis is perturbed [24]–[27]. We also confirmed that ricin degradation was not affected by CHC knockdown (not shown). In order to confirm the increased retrograde transport in CHC knockdown cells, we measured the mannosylation of ricin which takes place in the endoplasmic reticulum. In agreement with the sulfation data, we observed a strong increase in ricin mannosylation when CHC was knocked down (Figure 5c, black bars). Total protein mannosylation was also mildly increased at this condition (Figure 5c, white bars). We next studied the cellular localization of ricin by confocal microscopy. Interestingly, ricin appeared in dense perinuclear clusters in knockdown cells, in contrast to the spread vesicular localization in control cells (Figure 5d). Also SNX4 changed its localization upon CHC knockdown (Figures 5d and S3). Interestingly, it co-localized to a similar extent (∼40%) with ricin both in control and knockdown cells (Figure 5d), consistent with its role in ricin transport [9]. In knockdown cells, some CHC could still be detected close to the nucleus, and of this, 73.8±14.0% (average with standard deviation, n = 7) co-localized with SNX4. In agreement with the biochemical assays (Figure 5b, c), the ricin cluster in knockdown cells co-localized with the *trans*-Golgi protein TGN46 (Figure 5e). Quantification revealed a 2.4 fold increase in ricin co-localization with TGN46 in CHC knockdown cells compared to control cells, and a 22.6 fold increase in TGN46 structures positive for ricin (n = 4 for control cells and 7 for siCHC cells, p = 0.025 and <0.0001 by Student's *t*-test for the two measurements, respectively). The difference in these numbers is likely due to the fact that TGN46 labelling remained stable upon CHC knockdown, whereas ricin was often more easily detected in knockdown cells due to its clustering (see Figure 5e). This will influence the relative calculations. In summary, these data show that ricin transport to the Golgi apparatus is enhanced upon CHC knockdown. We also studied the effect of expressing the SNX4 109-113Δ mutant on ricin trafficking, but did not observe any clear effect (data not shown).

## Discussion

SNX4 binds to PI(3)P at the endosome, and is an effector of the PI3-kinase hVps34 in endosome to Golgi transport of ricin [9]. Furthermore, the activity of hVps34 is required for minus-end directed endosome motility [13], and SNX4 has recently been reported to interact with KIBRA, which binds the minus-end directed motor protein dynein [10]. In the present study, we confirm that SNX4 and dynein are found in the same complex, and show that SNX4 also binds CHC. However, the roles of dynein and CHC in endosome motility are contrary. CHC appears to serve as a negative regulator of vesicular movement. This is supported by both biochemical and imaging data showing that CHC knockdown leads to increased retrograde transport of ricin.

An interaction between SNX4 and CHC has not previously been reported. It is not clear why a proteomics study of clathrin coated vesicles in HeLa cells identified several SNXs, but not SNX4 [28]. The CHC interacting motif in SNX4 was in the present study identified to be a short clathrin box resembling sequence. This motif is part of an helical structure, α1, conserved in PX-domain containing proteins [29]. The sequence similarity to the PX domain in the crystallized p40^phox^ suggests that this helix is exposed at the protein surface also in SNX4 [30]. Amino acids directly upstream of the clathrin box resembling motif are involved in PI(3)P binding [30]. When the sequence was deleted, SNX4 co-localization with EEA1 was only slightly reduced, while binding to CHC was strongly inhibited. However, it was not completely abolished, indicating that another part of the protein may be involved in CHC binding. The same has been observed for epsin 1 [31] and Hrs [20], both containing a clathrin box. Still, by expressing the SNX4 clathrin box in a short peptide, we showed that it was sufficient to pull down CHC. Furthermore, individual amino acids in the motif were crucial for the CHC binding. Importantly, we cannot exclude that the mutation analysis abrogates the helical structure of the domain. Interestingly, we found that dynein interacted with the same region of SNX4 as CHC, but the clathrin box variant was not sufficient for dynein binding. For both CHC and dynein, the interaction was stronger with the longest SNX4 peptide (101–132), probably because this peptide contains the complete α1 helix [29]. PX domains of other proteins have also been shown to be involved in protein-protein interactions [29], [32], and we found that the corresponding sequences from SNX1, SNX2 and SNX3 also could bind CHC. A variety of clathrin boxes has previously been identified [31], [33], and we propose that the CHC interacting sequence of SNX4 functions as a clathrin box.

The role of CHC in ricin retrograde transport has previously been studied using a CHC antisense inducible cell line [34]. In this cell line, transport of ricin from endosomes to the Golgi was not affected [34]. An important difference between the antisense system and the siRNA system used in the present study is that the CHC protein level is reduced upon siRNA treatment. The antisense cell line still has CHC, although not functional, present at a high level [35]. Interestingly, a different requirement for CHC is reported for endosome to Golgi transport of Shiga toxin [36], [37]. Shiga toxin induces phosphorylation of CHC, which again regulates toxin uptake [38]. It is possible that such CHC phosphorylation is necessary for proper endosome to Golgi transport of Shiga as well.

We suggest that dynein access SNX4 after CHC release. This is in agreement with the findings that the dynein/SNX4 interaction is independent of CHC, and that dynein and CHC bind to the same region of SNX4. When cells were treated with nocodazole, an increased binding of CHC to SNX4 was observed. This could be a result of obstructed vesicle movement due to tubulin depolymerisation, and therefore SNX4 remains bound to CHC. Surprisingly, we did not observe an increased binding of dynein to SNX4 upon CHC knockdown. This may be explained by an altered kinetics in knockdown cells, since when CHC is reduced, endosomes appear closer to the Golgi apparatus [39], [40]. The finding that CHC binding to SNX4 is increased while dynein remains stable further indicates that CHC binding to SNX4 is independent of dynein. However, one can not exclude that dynein is present in excess and therefore sufficient for further recruitment of CHC. By showing that SNX4 exists in complex with CHC, most probably upstream of its interaction with dynein, we contribute with another piece of the endosomal puzzle.

## Supporting Information

Text S1 (0.04 MB DOC)

Figure S1 SNX4 co-localizes with GFP-CLC. Cells were transfected with GFP-CLC for 24 h before fixation and staining with anti-SNX4 antibodies. Bar, 10 µm.
(3.59 MB TIF)

Figure S2 Cellular localization of SNX4 mutants (a) Cells transfected with the indicated myc-tagged SNX4 constructs were fixed and stained with anti-myc antibodies. DRAQ5 was used to stain the nuclei. (b) Cells transfected with the indicated myc-tagged SNX4 constructs were fixed and stained with anti-myc and anti-EEA1 antibodies. Bars, 10 µm.
(0.26 MB PDF)

Figure S3 GFP-SNX4 alters its localization in CHC knockdown cells. Cells transfected with a control vector or siCHCv for 72 h were transfected with GFP-SNX4 for the last 24 h before fixation and staining as indicated. DRAQ5 was used to stain the nuclei. Bar, 5 µm.
(0.18 MB PDF)
