# Supplementary material for: SNX4 in Complex with Clathrin and Dynein: Implications for Endosome Movement
Source: PLoS One. 2009 Jun 16;4(6):e5935. doi: 10.1371/journal.pone.0005935 (PMC2691479; doi:10.1371/journal.pone.0005935)
Supplement: Text S1 — (0.04 MB DOC) [file pone.0005935.s001.doc]

**Text S1**

***GST-peptide cloning***

We designed primers encoding the reverse CHC box containing *Bam*HI and *Not*I overhangs (lower case). The expected protein product will contain the GST followed by Q (which comes from the framing of the restriction site to the sequence), the peptide sequence and AAAS. The primers used to construct the different GST-fusions were:

| **Primer name** | **Primer sequence (5’->3’)** | **Construct name** | **Peptide sequence** |
| --- | --- | --- | --- |
| GSTCBe_UP | gatccAACGGCGATATAGTGAATTTGAGTTGTTGAGAAGCTACgc | 105-116 | RRYSEFELLRSY |
| GSTCBe_DWN | ggccgcGTAGCTTCTCAACAACTCAAATTCACTATATCGCCGTTg |
| GSTCBL_UP | gatccAAGACTCACTATGGCGGCGATATAGTGAATTTGAGTTGTTGAGAAGCTACCTTTTAGTTTACTATCCACATATTGTTGTGCCACCTCTGCCAGAAAAAgc | 101-132 | DSLWRRYSEFELLRSYLLVYYPHIVVPPLPEK |
| GSTCBL_DWN | ggccgcTTTTTCTGGCAGAGGTGGCACAACAATATGTGGATAGTAAACTAAAAGGTAGCTTCTCAACAACTCAAATTCACTATATCGCCGCCATAGTGAGTCTTg |
| GSTCBe1_UP | gatccAACGGCGATATAGTGcATTTGAGTTGTTGAGAAGCTACgc | E109A | RRYSaFELLRSY |
| GSTCBe2_UP | gatccAACGGCGATATAGTGAAgcTGAGTTGTTGAGAAGCTACgc | F110A | RRYSEaELLRSY |
| GSTCBe3_UP | gatccAACGGCGATATAGTGAATTTGcGTTGTTGAGAAGCTACgc | E111A | RRYSEFaLLRSY |
| GSTCBe4_UP | gatccAACGGCGATATAGTGAATTTGAGgcGTTGAGAAGCTACgc | L112A | RRYSEFEaLRSY |
| GSTCBe5_UP | gatccAACGGCGATATAGTGAATTTGAGTTGgcGAGAAGCTACgc | L113A | RRYSEFELaRSY |

For the alaninie-scan, only the upper strand primers are shown (mutation in lower case underlines). “e” stands for elongated and “L” for Long.

The annealing of each strand was performed as follows: the two primers (named UP and DOWN) were diluted in annealing buffer (100 mM Tris pH 8.0, 50 mM NaCl, 1 mM EDTA) to a final concentration of 1 μM. The mix was boiled for 5 minutes at 95ºC and the heating block was left at room temperature for 1 hour. 2 μl of this mix (20 nM of annealed primer) was mixed with 1 μl (10 to 50 ng) of pGEX-5X (Pharmacia, Uppsala, Sweden), previously opened with *Bam*HI and *Not*I and gel-purified. 3 μl Mighty Mix (Takara, Otsu, Shiga, Japan) was added to the DNA, and the mix incubated for 10 minutes at room temperature. The ligation was finally transformed into XL1B competent cells (Stratagene, La Jolla, CA). Individual colonies were picked, and their DNA sequenced.
