# Supplementary figures and images for: SNX4 in Complex with Clathrin and Dynein: Implications for Endosome Movement
Source: PLoS One. 2009 Jun 16;4(6):e5935. doi: 10.1371/journal.pone.0005935 (PMC2691479; doi:10.1371/journal.pone.0005935)

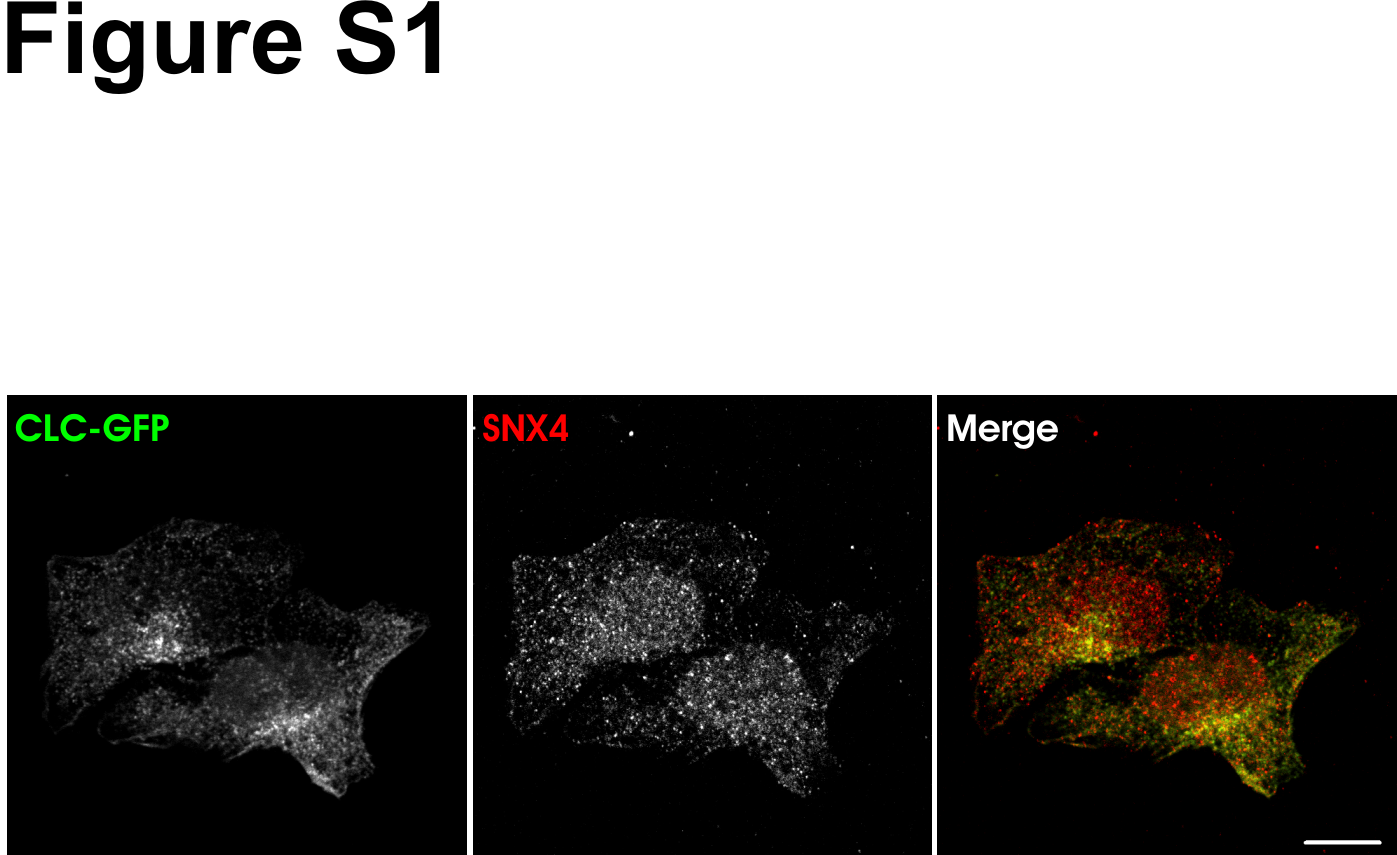

Supplement: Figure S1 — SNX4 co-localizes with GFP-CLC. Cells were transfected with GFP-CLC for 24 h before fixation and staining with anti-SNX4 antibodies. Bar, 10 µm. (3.59 MB TIF) [file pone.0005935.s002.tif]

# Figure S2

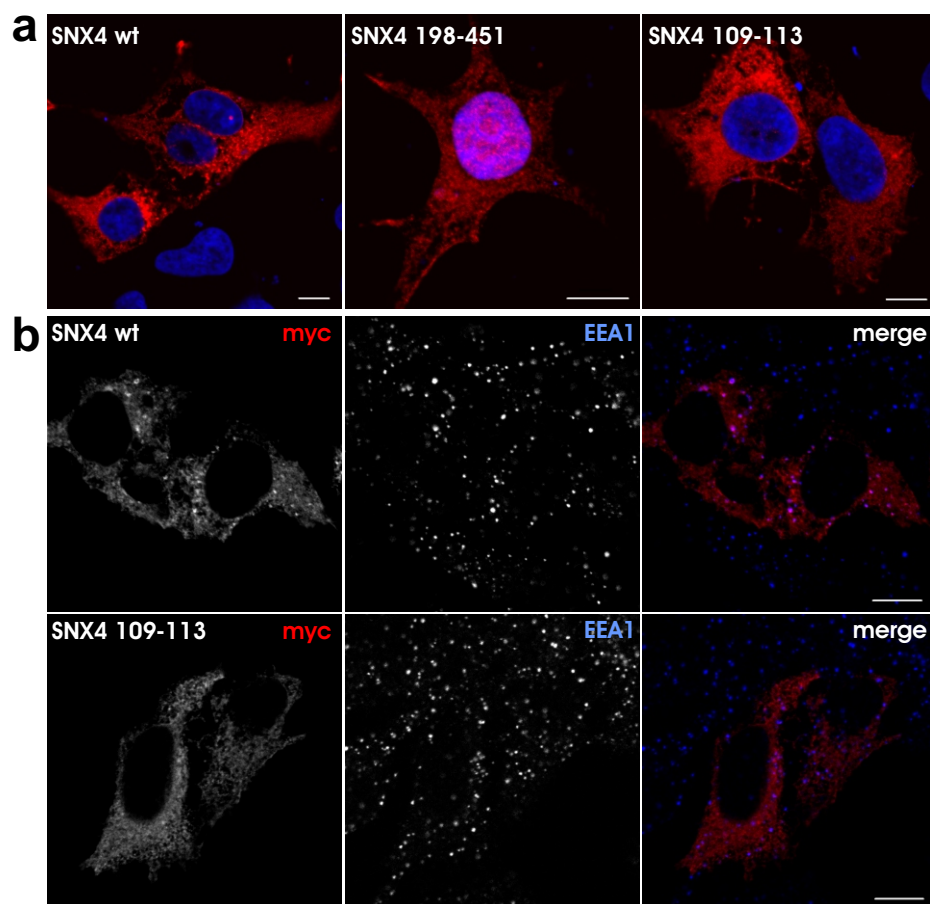

Supplement: Figure S2 — Cellular localization of SNX4 mutants (a) Cells transfected with the indicated myc-tagged SNX4 constructs were fixed and stained with anti-myc antibodies. DRAQ5 was used to stain the nuclei. (b) Cells transfected with the indicated myc-tagged SNX4 constructs were fixed and stained with anti-myc and anti-EEA1 antibodies. Bars, 10 µm. (0.26 MB PDF) [file pone.0005935.s003.pdf]

# Figure S3

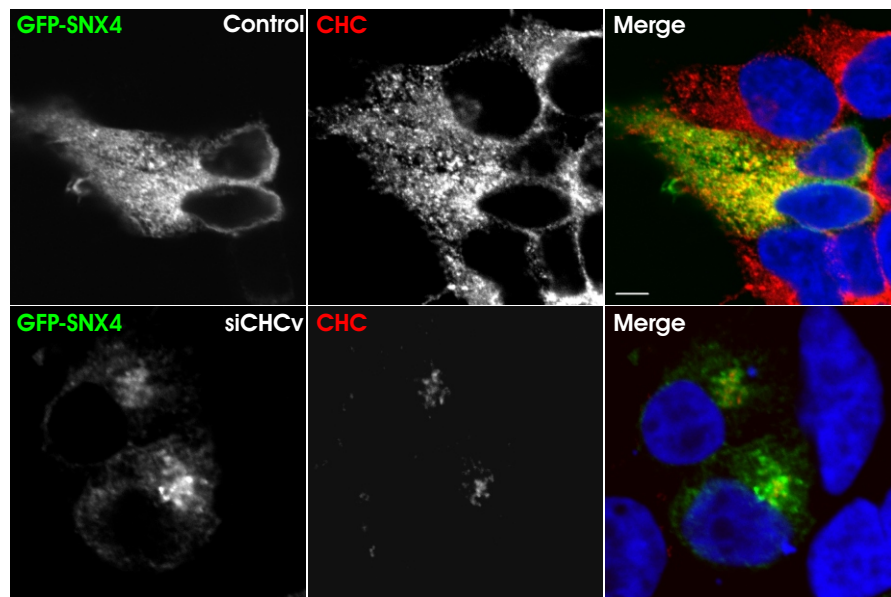

Supplement: Figure S3 — GFP-SNX4 alters its localization in CHC knockdown cells. Cells transfected with a control vector or siCHCv for 72 h were transfected with GFP-SNX4 for the last 24 h before fixation and staining as indicated. DRAQ5 was used to stain the nuclei. Bar, 5 µm. (0.18 MB PDF) [file pone.0005935.s004.pdf]
